# Association between *TIMP-2* gene polymorphism and breast cancer in Han Chinese women

**DOI:** 10.1186/s12885-019-5655-8

**Published:** 2019-05-14

**Authors:** Kai Wang, Guanying Wang, Shangke Huang, Anqi Luo, Xin Jing, Gang Li, Yi Zhou, Xinhan Zhao

**Affiliations:** 1 Department of Internal Medicine Oncology, The First Affiliated Hospital of Xi’an Jiaotong University, #277 West Yanta Road, Xi’an, 710061 Shaanxi China; 2 The Second Department of Spleen and Stomach, The Hospital of Traditional Chinese Medicine of Shaanxi Province, Xi’an, 710063 Shaanxi China; 3 The Second Department of Thoracic Surgery, The First Affiliated Hospital of Xi’an Jiaotong University, Xi’an, 710061 Shaanxi China; 4 The Center for Medical Imaging, The Hospital of Traditional Chinese Medicine of Shaanxi Province, Xi’an, 710063 Shaanxi China

**Keywords:** Breast cancer, *TIMP-2*, Gene polymorphism, Case-control study

## Abstract

**Background:**

TIMP-2 gene plays an important role in the development of breast cancer. The present study was conducted to evaluate whether *TIMP-2* gene polymorphisms are associated with breast cancer risk in a Han Chinese cohort.

**Methods:**

Six single nucleotide polymorphisms (SNPs) within the *TIMP-2* gene in 571 breast cancer and 578 healthy control subjects were genotyped through the Agena MassARRAY. Logistic regression analysis was used to assess the influence of *TIMP-2* polymorphisms on breast cancer. Functional annotation of *TIMP-2* variants and *TIMP-2* expression were analyzed by bioinformatics.

**Results:**

Bioinformatics analysis found that rs4789936 was likely to affect transcription factor binding, motifs, DNase footprint, and DNase peaks; and *TIMP-2* was under-expressed in breast cancer, the risk allele of rs4789936 was associated with increased expression of *TIMP-2* in peripheral blood samples. Importantly, individuals carrying *TIMP-2* rs2277698 T allele have a 19% lower risk of breast cancer than individuals with allele C, providing protection (OR = 0.81, 95%CI = 0.67–0.99, *p* = 0.041). In the breast cancer patients with c-erb positive and PR positive, when the CC genotype was used as a reference, individuals carrying the TT genotype increased the risk of breast cancer. Haplotype analysis showed “TCC” was associated with a reduced risk of breast cancer (OR = 0.79, 95%CI = 0.63–0.97, *p* = 0.028).

**Conclusion:**

Our study indicated that *TIMP-2* rs2277698 was associated with breast cancer susceptibility.

## Background

As one of the most prevalent malignancies with highly invasive and metastatic potential, breast cancer continues to be a major global health concern that leads to increasing morbidity and mortality among women worldwide [1]. Domestic and foreign scholars believe that extracellular matrix (ECM) plays a vital role in the invasion and migration of breast cancer cells [2]. Additionally, these studies have demonstrated that degradation of the basement membrane ECM is critical for the progression of tumorigenesis and metastasis [3]. Matrix metalloproteinase-2 (MMP-2) degrades type IV collagen, which is one of the major structural components of the basement membrane ECM. Based on this function, MMP-2 is considered a crucial enzyme in the regulation of tumor proliferation and metastasis [4]. Previous studies have shown that *MMP-2* expression is elevated in cancer patients compared with control subjects and is associated with advanced stages of disease and worse prognosis [5].

Tissue inhibitor of metalloproteinase-2 (TIMP-2) is an endogenous inhibitor of MMP-2 that has been implicated in the regulation of MMP-2 proteolytic activity through formation of a 1:1 stoichiometric inhibitory complex with the enzyme [6]. Genetic polymorphisms in the *TIMP-2* gene, located on chromosome 17q25, may lead to an increase or decrease in TIMP-2 activity and subsequently disrupt the balance between the activity of TIMP-2 and MMP-2*.* This disrupted balance could then influence cancer development and progression [7]. More and more research have shown that *TIMP-2* mutation influence the risk of the development and persistence of numerous carcinomas and diseases [8–12]. The correlation between the genetic variants of *TIMP-2* and susceptibility to stroke [13], oral squamous cell carcinoma [8], prostate cancer [9], abdominal aortic aneurysm [10], head and neck squamous cell carcinoma [11], and gastric cancer [12] have been identified in a number of studies worldwide. Taken together, these findings suggest that evaluation of *TIMP-2* polymorphism in cancers may be useful as a prognostic indicator.

Very few studies have evaluated polymorphism of *TIMP-2* in individuals with breast cancer. Combining with the existing literature reports, and minor allele frequencies (MAFs) of greater than 5% in the global population, we selected rs2277698, rs2009196, rs7342880, rs11654470, rs2003241, and rs4789936 six SNPs to research the effect of *TIMP-2* gene polymorphisms on the susceptibility of breast cancer in a cohort of Han Chinese women. Genetic screening involving polymorphism of the *TIMP-2* gene could provide valuable information for breast cancer susceptibility and identification of high risk patients.

## Methods

### Study participants

From the First Affiliated Hospital of Xi’an Jiaotong University, we recruited 571 breast cancer patients (mean age: 50.91 ± 11.23 years), which were recently diagnosed, histologically confirmed, presented without any previous acute or chronic pathology. We also recorded some clinical information about patients from the patients’ medical records, as shown in Table 1. Consist of smoking and drink status, tumor size, clinical stages, Lymph node metastasis (Yes, or No), menopausal status (Yes, or No), procreative times, estrogen receptor (ER) status (Positive or negative), progesterone receptor (PR) status (Positive or negative), and c-erbB status (Positive or negative). At the same time 578 healthy subjects (mean age: 49.22 ± 10.11 years) were recruited from a large cohort of Han Chinese women, the Controls were generally healthy without diseases related to the vital organs.

**Table 1:** The characteristics of breast cancer cases and cancer-free controls

Characteristics	Cases	Control	*P* value
Number	566	578	
Age (mean ± SD)	50.91 ± 11.23	49.22 ± 10.11	0.017
smoking and drink			
No	328		
Yes	40		
Age of menarche			
≤14	145		
>14	119		
Menopausal status			
Premenopausal	104		
Postmenopausal	156		
Procreative times			
<1	104		
≥1	130		
Tumor size			
<2 cm	105		
≥2 cm	154		
Tumor site			
left	256		
right	241		
Lymph node involvement			
Negative	201		
Positive	197		
Clinical stage			
III-IV	114		
I- II	272		
Immunohistochemistry results			
ER (−)	108		
ER (+)	208		
PR (−)	145		
PR (+)	166		
c-erb(−)	91		
c-erb(+)	214		

*ER* Estrogen receptor, *PR* Progesterone receptor

### SNP selection and genotyping

We selected the GoldMag-Mini Whole Blood Genomic DNA Purification Kit (GoldMag Co. Ltd. Xi’an City, China) to extract the DNA from the 5 ml peripheral venous blood; and Nanodrop 2000 (Gene Company Limited) was used to detect the concentration and purity of samples, DNA to ensure that the samples could be used for subsequent experiments. Same as previously published articles [14, 15]. rs2277698, rs2009196, rs7342880, rs11654470, rs2003241, and rs4789936 Six SNPs were selected in our study based on minor allele frequency data more than 0.05 in the global population [16]. Primer design and SNP typing were performed in the same way as previously published articles [14, 15]. The genotyping primers were designed with the Agena MassARRAY Assay Design 3.0 Software [17]. The Agena MassARRAY RS1000 was used for genotyping, and the related data were managed using Agena Typer 4.0 Software [13, 17, 18].

### Bioinformatics and expression analyses

To determine the effect of *TIMP-2* SNPs on chromatin structure and allele-specific transcription factor binding, we used RegulomeDB [19] and HaploReg V4 [20]. The effect of mutation on *TIMP-2* gene expressions in whole blood samples were further analyzed via the GTEX database (https://gtexportal.org/home/). Additionally, the UALCAN database [21] was used to analyze the expression of *TIMP-2* in breast cancer tissues and normal tissues.

### Statistical analysis

Demographic characteristics were counted. The Hardy-Weinberg equilibrium (HWE) was calculated by χ2 test [22]. Five genetic models were used to evaluate the association between gene polymorphisms and breast cancer risk. Odds ratios (ORs) and its corresponding 95%CI were estimated using an logistic regression model with adjustments for age and gender through the PLINK software [23]. Further analysis to assess the impact of polymorphism on breast cancer based on tumor size, lymph node metastasis, ER/PR/ c-erb status, histological grade, procreative times, age of menarche and menopausal status. Linkage disequilibrium among polymorphic sites was assessed with Haploview [24], and associations between haplotypes and breast cancer risk were analyzed with PLINK version 1.07 software. The threshold of *p* was set to 0.05.

## Results

Using RegulomeDB (Table 2), we found that rs4789936 was likely to affect transcription factor binding, motifs, DNase footprint, and DNase peaks. Additionally, rs2003241 was likely to affect transcription factor binding, motifs, and DNase peaks; whereas, the remaining genetic variants (rs2009196, rs7342880, and rs11654470) were only likely to affect transcription factor binding or DNase peaks. Consistent with these findings, HaploReg also predicted that rs2009196, rs7342880, rs1165447, rs2003241, and rs4789936 may result in motif changes (Table 2).

**Table 2:** Functional annotation of *TIMP-2* SNPs using RegulomeDB and HaploReg

SNP	Gene	Allele	RegulomeDB	HaploReg
rs2277698 (synonymous)	*TIMP-2*	T/C	No Data	SiPhy conse, Selected eQTL hits
rs2009196 (intronic)	*TIMP-2*	C/G	5	DNase, Motifs changed, Selected eQTL hits
rs7342880 (intronic)	*TIMP-2*	A/C	5	DNase, Motifs changed, Selected eQTL hits
rs11654470 (intronic)	*TIMP-2*	C/T	5	DNase, Motifs changed, Selected eQTL hits
rs2003241 (intronic)	*TIMP-2*	C/T	3a	DNase, Motifs changed, Selected eQTL hits
rs4789936 (5′-UTR)	*TIMP-2*	T/C	2b	Motifs changed, Selected eQTL hits

SNP: single nucleotide polymorphism; eQTL: expression quantitative trait loci; 2b: Transcription factor binding +any motif +DNase footprint + DNase peak; 3a: Transcription factor binding +any motif + DNase peak; 5: Transcription factor binding or DNase peak

Table 3 shows the location, alleles of the *TIMP-2* gene polymorphisms in the breast cancer group and the control group, and whether these sites satisfy the Hardy Weinberg equilibrium. Based on their deviation from HWE, rs11654470 and rs2003241 were excluded from the subsequent analyses. Importantly, the frequencies of the rs2277698 alleles were significantly different between breast cancer patients and control subjects, individuals carrying allele T have a 19% lower risk of breast cancer than individuals with allele C, providing protection (OR = 0.81, 95%CI = 0.67–0.99, *p* = 0.041).

**Table 3:** Basic characteristics and allele frequencies of the six SNPs in the *TIMP-2* gene

SNP	Gene	chromosome	Position	Allele	Minor allele frequency		HWE *p* value	OR (95%CI)	*p*
					Case	Control			
rs2277698	*TIMP-2*	17q25.3	76,867,017	T/C	0.201	0.236	0.0651	0.81(0.67–0.99)	0.041^*^
rs2009196	*TIMP-2*	17q25.3	76,870,581	C/G	0.392	0.426	0.3502	0.87(0.74–1.03)	0.099
rs7342880	*TIMP-2*	17q25.3	76,874,512	A/C	0.161	0.150	0.7433	1.09(0.87–1.37)	0.448
rs11654470	*TIMP-2*	17q25.3	76,877,331	C/T	0.23	0.273	0.0119^*^	0.80(0.66–0.96)	0.019
rs2003241	*TIMP-2*	17q25.3	76,885,117	C/T	0.164	0.161	0.0196^*^	1.02(0.82–1.28)	0.853
rs4789936	*TIMP-2*	17q25.3	76,897,974	T/C	0.299	0.307	0.6256	0.96(0.80–1.15)	0.658

SNP: single nucleotide polymorphism; OR: odds ratio; 95%CI: 95% confidence interval; HWE: Hardy-Weinberg equilibrium

*p < 0.05 indicates statistical significance

The detailed findings of the logistic regression analysis for each genetic model are presented in Table 4. Of note, we observed that the frequency of the heterozygous variant C/T genotype of *TIMP-2* rs2277698 was significantly reduced in breast cancer patients, when compared with healthy group. In the dominant model, after adjustment for age, the individuals with *TIMP-2* rs2277698 CT + TT genotype have a 24% lower risk of developing breast cancer than CC genotype (OR = 0.76, 95%CI = 0.60–0.97, *p* = 0.025).

**Table 4:** *TIMP-2* SNP genotypes and the risk of breast cancer based on the results of logistic regression model analysis

SNP	Model	Genotype	Control	Case	OR (95%CI)	*p*
rs2277698	Co-dominant	CC	329 (56.9%)	361 (63.3%)	1	0.080
		CT	225 (38.9%)	189 (33.2%)	0.77 (0.60–0.98)	
		TT	24 (4.2%)	20 (3.5%)	0.72 (0.39–1.33)	
	Dominant	CC	329 (56.9%)	361 (63.3%)	1	0.025*
		CT + TT	249 (43.1%)	209 (36.7%)	0.76 (0.60–0.97)	
	Recessive	CC + CT	554 (95.8%)	550 (96.5%)	1	0.460
		TT	24 (4.2%)	20 (3.5%)	0.80 (0.43–1.46)	
	Log-additive	–	–	–	0.80 (0.65–0.98)	0.029*
rs2009196	Co-dominant	GG	184 (31.9%)	202 (35.4%)	1	0.190
		CG	293 (50.9%)	290 (50.8%)	0.90 (0.69–1.16)	
		CC	99 (17.2%)	79 (13.8%)	0.72 (0.50–1.03)	
	Dominant	GG	184 (31.9%)	202 (35.4%)	1	0.210
		CG + CC	392 (68.1%)	369 (64.6%)	0.85 (0.67–1.09)	
	Recessive	GG + CG	477 (82.8%)	492 (86.2%)	1	0.100
		C/C	99 (17.2%)	79 (13.8%)	0.76 (0.55–1.05)	
	Log-additive	–	–	–	0.86 (0.72–1.02)	0.078
rs7342880	Co-dominant	CC	419 (72.5%)	399 (69.9%)	1	0.470
		AC	145 (25.1%)	160 (28.0%)	1.17 (0.90–1.52)	
		AA	14 (2.4%)	12 (2.1%)	0.90 (0.41–1.97)	
	Dominant	CC	419 (72.5%)	399 (69.9%)	1	0.300
		AC + AA	159 (27.5%)	172 (30.1%)	1.15 (0.89–1.48)	
	Recessive	CC + AC	564 (97.6)	559 (97.9)	1	0.710
		AA	14 (2.4%)	12 (2.1%)	0.86 (0.39–1.88)	
	Log-additive	–	–	–	1.10 (0.88–1.38)	0.410
rs4789936	Co-dominant	CC	280 (48.4%)	280 (49.0%)	1	0.850
		AC	241 (41.7%)	241 (42.2%)	1.00 (0.78–1.27)	
		AA	57 (9.9%)	50 (8.8%)	0.89 (0.59–1.35)	
	Dominant	CC	280 (48.4%)	280 (49.0%)	1	0.840
		AC + AA	298 (90.1%)	291 (51.0%)	0.98 (0.77–1.23)	
	Recessive	CC + AC	521 (90.1%)	521 (91.2%)	1	0.560
		AA	57 (9.9%)	50 (8.8%)	0.89 (0.60–1.33)	
	Log-additive	–	–	–	0.96 (0.81–1.15)	0.680

SNP: single nucleotide polymorphism; OR: odds ratio; 95%CI: 95% confidence interval

*p < 0.05 indicates statistical significance

As shown in Table 5, in the breast cancer patients with c-erb positive and PR positive, when the TIMP-2 rs2277698 CC genotype was used as a reference, individuals carrying the TT genotype promoted the risk of breast cancer by 72 and 63% in allele model, respectively (c-erb positive: OR = 1.72, 95%CI: 1.08–2.74, *p* = 0.022; PR positive: OR = 1.63, 95%CI: 1.09–2.43, *p* = 0.017). When less than 49 years old, individuals with TT genotype had a 31% lower risk of breast cancer than the CC genotype individuals (OR = 0.69, 95%CI: 0.52–0.9, *p* = 0.007).

**Table 5:** The associations between the *TIMP-2* rs2277698 polymorphism and clinical characteristics of BC patients

Variants	CC/TC/TT	Allele model		Genotype model				Dominant model		Recessive model		Additive model	
		OR (95%CI)	*p*	TC genotype OR (95%CI)	*p*	TT genotype OR (95%CI)	*p*	OR (95%CI)	*p*	OR (95%CI)	*p*	OR (95%CI)	*p*
c-erb													
Negative	126/76/11	1											
Positive	66/23/2	1.72(1.08–2.74)	0.022	1.78(1.02–3.1)	0.043	2.88(0.62–13.4)	0.178	1.87(1.09–3.2)	0.023	2.4(0.52–11.08)	0.261	1.75(1.09–2.82)	0.021
ER													
Negative	76/28/4	1											
Positive	124/74/9	1.43(0.93–2.19)	0.1	1.63(0.96–2.75)	0.069	1.38(0.41–4.63)	0.604	1.6(0.97–2.63)	0.068	1.18(0.36–3.94)	0.784	1.43(0.93–2.2)	0.102
PR													
Negative	102/39/4	1											
Positive	95/61/9	1.63(1.09–2.43)	0.017	1.66(1.01–2.71)	0.045	2.42(0.72–8.14)	0.152	1.73(1.08–2.78)	0.024	2.06(0.62–6.85)	0.239	1.62(1.08–2.43)	0.021
Clinical stage													
I- II	175/86/11	1											
III-IV	71/38/4	1.03(0.7–1.52)	0.874	1.09(0.5–2.35)	0.83	0.55(0.08–3.84)	0.542	1.01(0.48–2.13)	0.973	0.53(0.08–3.65)	0.518	0.94(0.5–1.76)	0.843
Menopausal status													
Premenopausal	64/34/6	1											
Postmenopausal	99/51/6	0.89(0.58–1.37)	0.598	1.09(0.5–2.35)	0.83	0.55(0.08–3.84)	0.542	1.01(0.48–2.13)	0.973	0.53(0.08–3.65)	0.518	0.94(0.5–1.76)	0.843
Lymph node involvement													
Negative	129/64/8	1											
Positive	127/61/7	0.96(0.67–1.36)	0.812	0.96(0.63–1.48)	0.861	0.9(0.32–2.55)	0.837	0.95(0.63–1.44)	0.827	0.91(0.32–2.56)	0.854	0.96(0.67–1.36)	0.803
≤49													
control	153/117/16	1											
case	199/98/11	0.69(0.52–0.9)	0.007	0.66(0.47–0.93)	0.017	0.5(0.23–1.13)	0.095	0.64(0.46–0.89)	0.008	0.59(0.27–1.31)	0.193	0.68(0.51–0.9)	0.007
>49													
control	176/108/8	1											
case	161/88/8	0.94(0.7–1.26)	0.684	0.89(0.62–1.27)	0.516	1.1(0.4–3)	0.852	0.9(0.64–1.28)	0.566	1.15(0.42–3.11)	0.785	0.94(0.69–1.27)	0.67
Procreative times													
≥1	79/43/8	1											
<1	64/37/3	0.89(0.57–1.38)	0.599	1.05(0.6–1.83)	0.869	0.48(0.12–1.93)	0.3	0.96(0.56–1.65)	0.889	0.47(0.12–1.87)	0.283	0.89(0.56–1.4)	0.611
Age of menarche													
>14	71/43/5	1											
≤14	93/45/7	0.89(0.59–1.36)	0.591	0.79 (0.47–1.33)	0.372	1.11(0.33–3.66)	0.868	0.82(0.5–1.36)	0.441	1.2(0.37–3.92)	0.76	0.89(0.59–1.36)	0.592
Tumor size													
≥2 cm	70/31/4	1											
<2 cm	90/56/8	1.34(0.86–2.07)	0.191	1.44(0.84–2.48)	0.189	1.5(0.43–5.25)	0.529	1.45(0.86–2.44)	0.166	1.32(0.38–4.56)	0.661	1.35(0.86–2.1)	0.188

*p < 0.05 indicates statistical significance

**Fig. 1:**
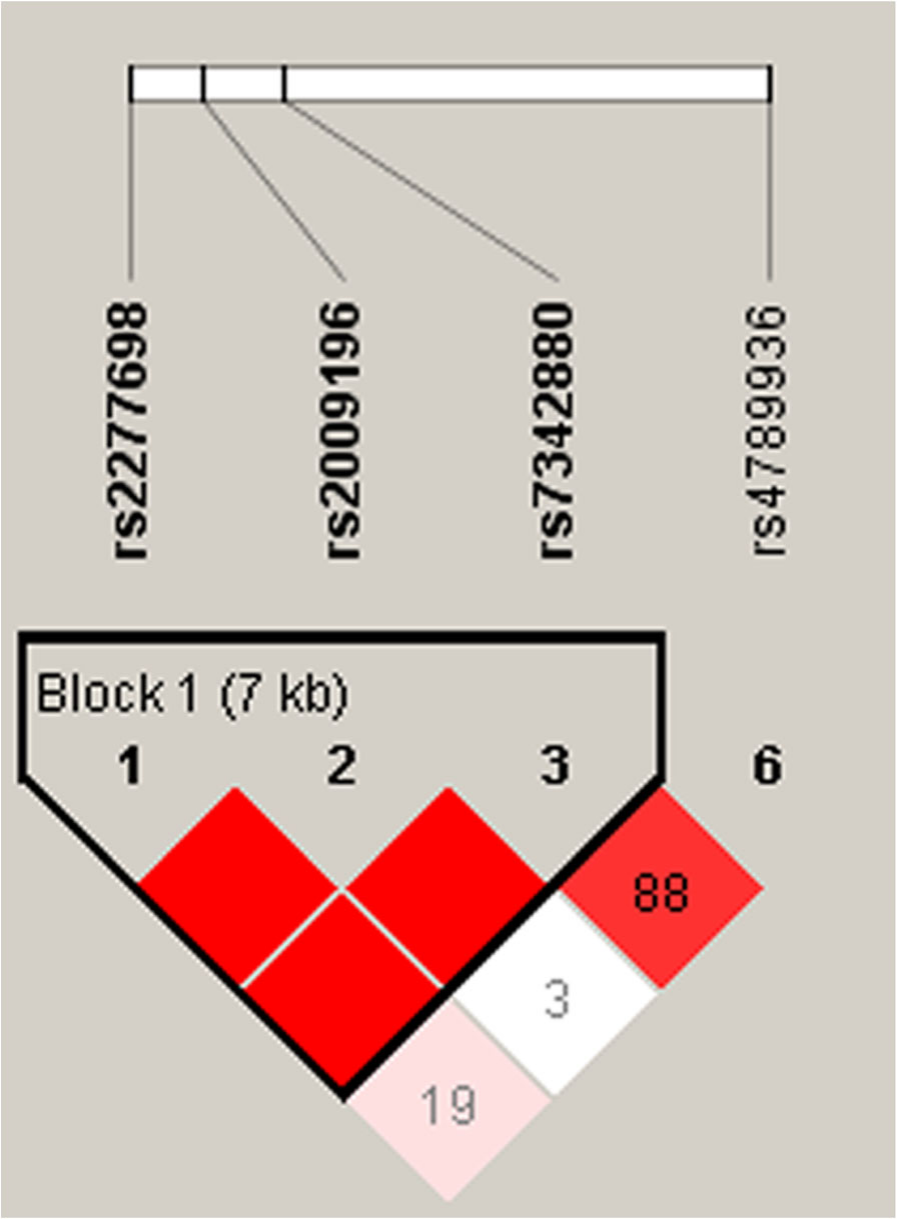
Haplotype block map for the six SNPs in the *TIMP-2* gene. Block 1 includes rs2277698, rs2009196, and rs7342880 with D′ = 1 (100%) for the corresponding variants

Linkage analysis indicated that rs2277698, rs2009196, and rs7342880 exhibit extremely significant linkage disequilibrium (Fig. 1). Therefore, the haplotype frequencies of these SNPs were further examined for association with breast cancer (Table 6). Indeed, when the haplotype “CGC” used as a reference, the haplotype “TCC” was associated with a reduce ed. risk of breast cancer (OR = 0.79, 95%CI = 0.63–0.97, *p* = 0.028).

**Table 6:** *TIMP-2* haplotype frequencies and the association with breast cancer

SNP	Haplotype	Freq	OR (95%CI)	*p*
rs2277698|rs2009196 |rs7342880	CGC	0.591	1	
	TCC	0.218	0.79 (0.63–0.97)	0.028^*^
	CCA	0.155	1.03 (0.81–1.30)	0.830
	CCC	0.036	0.64 (0.41–1.02)	0.059

SNP: single nucleotide polymorphism; OR: odds ratio; 95%CI: 95% confidence interval

*p < 0.05 indicates statistical significance

**Fig. 2:**
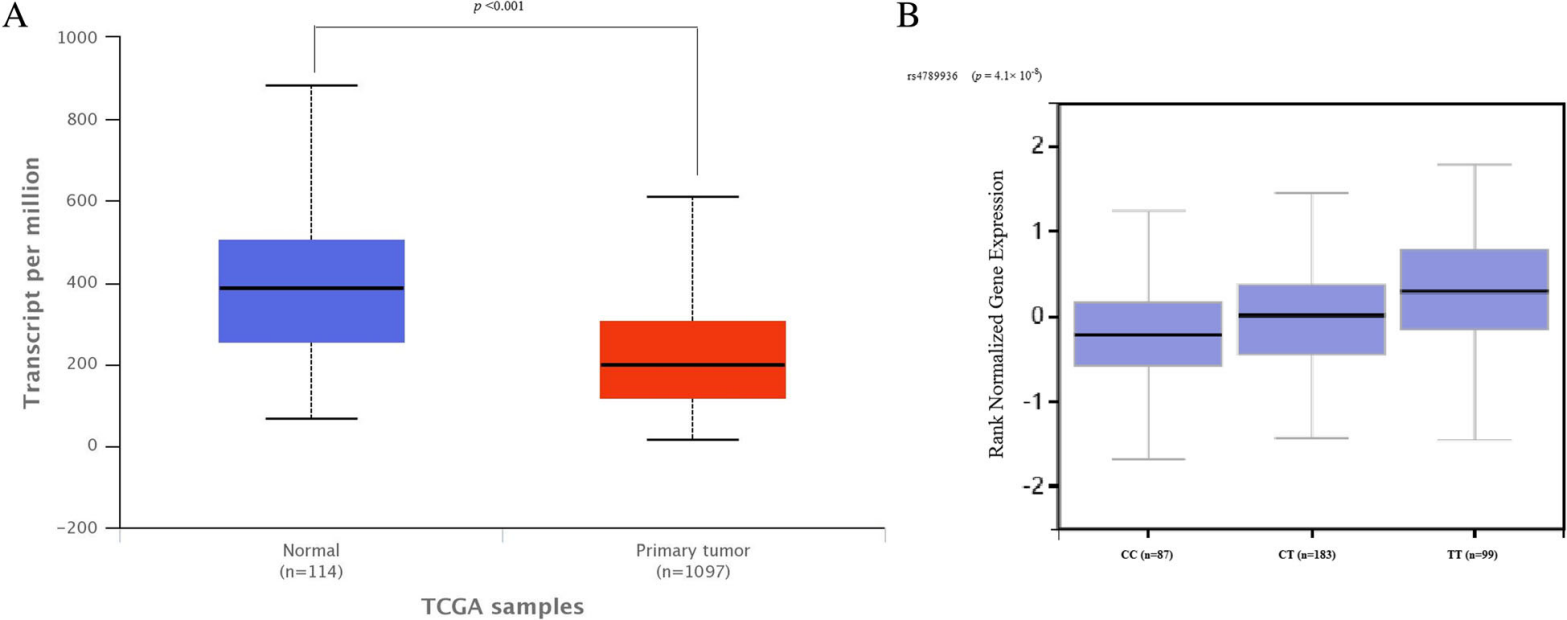
Expression of *TIMP-2* in human tissue databases. A: *TIMP-2* gene expression is down-regulated in breast cancer (*n* = 1097) compared with normal tissues (*n* = 144). B: Expression quantitative trait loci (eQTL) analyses of rs4789936 with *TIMP-2* mRNA expression levels in whole blood samples

To further validate our findings, we employed the use of two publically-available data sets. Examination of 1097 breast cancer tissues and 114 normal tissues from The Cancer Genome Atlas (TCGA) using the UALCAN database demonstrated that *TIMP-2* was under-expressed in breast cancer tissues (Fig. 2a). The GTEx database shown that the expression level of carrying the TT genotype is higher than that of the individual carrying the CC genotype,the risk allele of rs4789936 was associated with increased expression of *TIMP-2* (*p* = 4.1× 10^− 8^) in peripheral blood samples (Fig. 2b).

## Discussion

In this study, we found that SNP rs2277698 and haplotype, “TCC” in *TIMP-2* was significantly associated with an altered risk of breast cancer. Additionally, the UALCAN database demonstrated that the *TIMP-2* gene was under-expressed in breast cancer tissues. Based on the GTEx portal, the rs4789936 risk allele “A” increased the expression of *TIMP-2* in peripheral blood samples.

In the context of tumor invasion, TIMP-2 is expected to serve as an anti-invasive/anti-metastatic agent through inhibition of MMP-2. Changes in the level of TIMP-2 are known to directly affect the activity level of MMP-2 [25]. In addition, experimental evidence indicates that TIMP-2 has pleiotropic activities, including inhibition of endothelial cell growth induced by basic fibroblast growth factor, suppression of angiogenesis, and regulation of apoptosis [26]. Our analysis using the UALCAN database showed that the *TIMP-2* gene was under-expressed in breast cancer tissues. A common polymorphism in the *TIMP-2* gene is the C to T transition at position 303 (C303T, rs2277698), which results in a synonymous amino acid change at codon position 101 (Ser101Ser). *TIMP-2* gene mutation is associated with the occurrence of multiple diseases, including alcohol induced osteonecrosis of the femoral head [27], emphysema and paraseptal emphysema [28], and gastric cancer [29]. One research explore the association between TIMP-2 and breast cancer, and found that TIMP-2 rs7501477 and rs8064344 mutation affects the genetic susceptibility of breast cancer; while, no effect of rs2277698 mutation on breast cancer was found [16]. In Korean women Primary ovarian insufficiency (POI), revealed that TIMP-2 rs817990 GC (OR = 0.581) genotype and rs2277698 AA-GA (OR = 1.559) genotype influence the risk of Primary ovarian insufficiency in Korean women [30]. However, in our study, we only observed that rs2277698 mutation was associated with genetic susceptibility to breast cancer, and in the breast cancer patients with c-erb positive and PR positive, individuals carrying the TT genotype increased the risk of breast cancer. No other significant results were found. Combined with existing reports, we believe that rs2277698 is a susceptibility site for breast cancer, even affecting gynecological diseases. Other people reported significant results which we did not find in this study, which may be due to the false negative results caused by our small sample size. rs2277698 AA-GA (OR = 1.559) genotype influence the risk of Primary ovarian insufficiency in Korean women, while in our research our, rs2277698“T” allele with decreased breast cancer risk, this may be due to different functions of the same locus in different diseases and genetic differences among populations. Linkage disequilibrium analysis shown that rs2277698 was strongly linked to rs9889410 and rs11654470 in the 1000 Genomes Project population (r^2^ > 0.9), Bioinformatics analysis found that some of which (rs9889410 and rs11654470) reside in a region may be involved in changing transcriptional regulation [31]. Therefore, we speculate that rs2277698 may affect the transcription rate of *TIMP-2*. However, additional studies are necessary to validate these findings, and the protective mechanism of rs2277698 requires further investigation by biological means.

In our research, we found that rs7342880 and rs4789936 in *TIMP-2* gene have no effect on the genetic susceptibility of breast cancer. Nevertheless, a previous study suggested that mutations in the rs4789936 locus not only affect the genetic susceptibility of breast cancer, but also affect the survival of breast cancer patients [16]. So, the role of rs4789936 mutation on the genetic susceptibility of breast cancer remains controversial. Bioinformatics analysis found that mutation of the rs4789936 locus affects the expression of the *TIMP-2* gene in peripheral blood samples, the expression level of carrying the TT genotype is higher than that of the individual carrying the CC genotype. And, *TIMP-2* was under-expressed in breast cancer tissues. So, we will first expand the sample size to verify whether mutations at this site will affect the risk of breast cancer, and further explore how mutations at this site affect breast cancer development through functional tests.

Although some clinical indicators were collected in this study and stratified analysis was performed, the sample size of complete clinical information was small, which made some indicators unable to be analyzed hierarchically, for example, obesity, smoking and drinking. We will continue to refine this information for in-depth analysis.

## Conclusions

In conclusion, this study suggests that the *TIMP-2* rs2277698 polymorphism is associated with breast cancer in Han Chinese women, and the individuals that carry the CT genotype and “TCC” haplotype may be at reduced risk for breast cancer. Future investigation should focus on studies using large sample sizes or establish breast cancer cell lines that further explore how mutations at this site affect breast cancer development through functional tests.

## Abbreviations

95%CI: 95% confidence interval
ER: estrogen receptor
GTEx: Genotype-Tissue Expression
HWE: Hardy-Weinberg equilibrium
LD: linkage disequilibrium
OR: odds ratio
PR: progesterone receptor
SNPs: single nucleotide polymorphisms
TIMP-2: tissue inhibitor of metalloproteinase-2
